# Mast cell density in Merkel cell carcinoma and its correlation with prognostic features and MCPyV status: a pilot study

**DOI:** 10.1007/s10238-024-01366-4

**Published:** 2024-07-05

**Authors:** Gerardo Cazzato, Roberto Tamma, Margherita Fanelli, Anna Colagrande, Andrea Marzullo, Eliano Cascardi, Irma Trilli, Loredana Lorusso, Teresa Lettini, Giuseppe Ingravallo, Domenico Ribatti

**Affiliations:** 1 Section of Molecular Pathology, Department of Precision and Regenerative Medicine and Ionian Area (DiMePRe-J), University of Bari “Aldo Moro”, 70124 Bari, Italy; 2 Department of Translational Biomedicine and Neuroscience, Section of Human Anatomy and Histology, University of Bari Medical School, Bari, Italy; 3 Department of Interdisciplinary Medicine, University of Bari “Aldo Moro”, 70124 Bari, Italy

**Keywords:** Merkel cell carcinoma, Tumor microenvironment, Mast cells, Tryptase, Recurrency, MCPyV

## Abstract

Merkel cell carcinoma (MCC) is a rare, highly aggressive, primitive neuroendocrine carcinoma of the skin, the origin of which is not yet fully understood. Numerous independent prognostic factors have been investigated in an attempt to understand which are the most important parameters to indicate in the histological diagnostic report of MCC. Of these, mast cells have only been studied in one paper before this one. We present a retrospective descriptive study of 13 cases of MCC, received at the Department of Pathology over a 20-year period (2003–2023 inclusive) on which we performed a study using whole-slide (WSI) morphometric analysis scanning platform Aperio Scanscope CS for the detection and spatial distribution of mast cells, using monoclonal anti-tryptase antibody and anti-CD34 monoclonal antibody to study the density of microvessels. In addition, we analyzed MCPyV status with the antibody for MCPyV large T-antigen (Clone CM2B4). We found statistically significant correlation between mast cell density and local recurrence/distant metastasis/death-of-disease (*p* = 0.008). To our knowledge, we firstly reported that MCPyV ( −) MCC shows higher mast cells density compared to MCPyV ( +) MCC, the latter well known to be less aggressive. Besides, the median vascular density did not show no significant correlation with recurrence/metastasis/death-of-disease, (*p* = 0.18). Despite the small sample size, this paper prompts future studies investigating the role of mast cell density in MCC.

## Introduction

Merkel cell carcinoma (MCC) is a rare, aggressive, neuroendocrine carcinoma of the skin, first described by Toker in 1972 and is also referred as trabecular carcinoma, Toker’s tumor or primitive neuroendocrine carcinoma of the skin [1]. In the latest classification of skin tumors by the World Health Organization (WHO) [2], MCC is defined by ICD-O code 8247/3, and epidemiologically, it is more frequent in the white population, with a mean age at diagnosis of 75 years and <5% of diagnosed patients under 50 years of age [3].

The number of reported cases of MCC has increased in recent times, the cell of origin of MCC is still debated, and in terms of etiopathogenesis, a certain number of MCC is linked to the presence of the Merkel cell polyomavirus (MCPyV) infection, as first discovered in 2008 by Feng et al. [4], who, using digital transcriptome subtraction, succeeded in elucidating the molecular pathways of virus-related oncogenesis of MCC. Currently, MCPyV is considered to be responsible for about 80% of MCC cases, with the remaining cases (about 20%) presenting a high mutational burden with prominent UV-related signatures in cellular DNA [5]. Regarding this latter type of MCC, the most frequent mutations are of the tumor suppressor genes RB1 and TP53 but also mutations promoting the activation of the PI3K pathway (HRAS and KRAS) and inactivation of the Notch pathway that is another critical oncogenic event [6]. In terms of topography, MCC usually favors the head–neck region (50%) (particularly the eyelid and periorbital region) and upper extremities (40%) and often presents as a firm, painless, nodular or plaque-like, flesh-colored to red–violet lesion, which can sometimes grow very rapidly to the point of causing ulceration, bleeding, and necrosis [5, 7, 8].

**Fig. 1:**
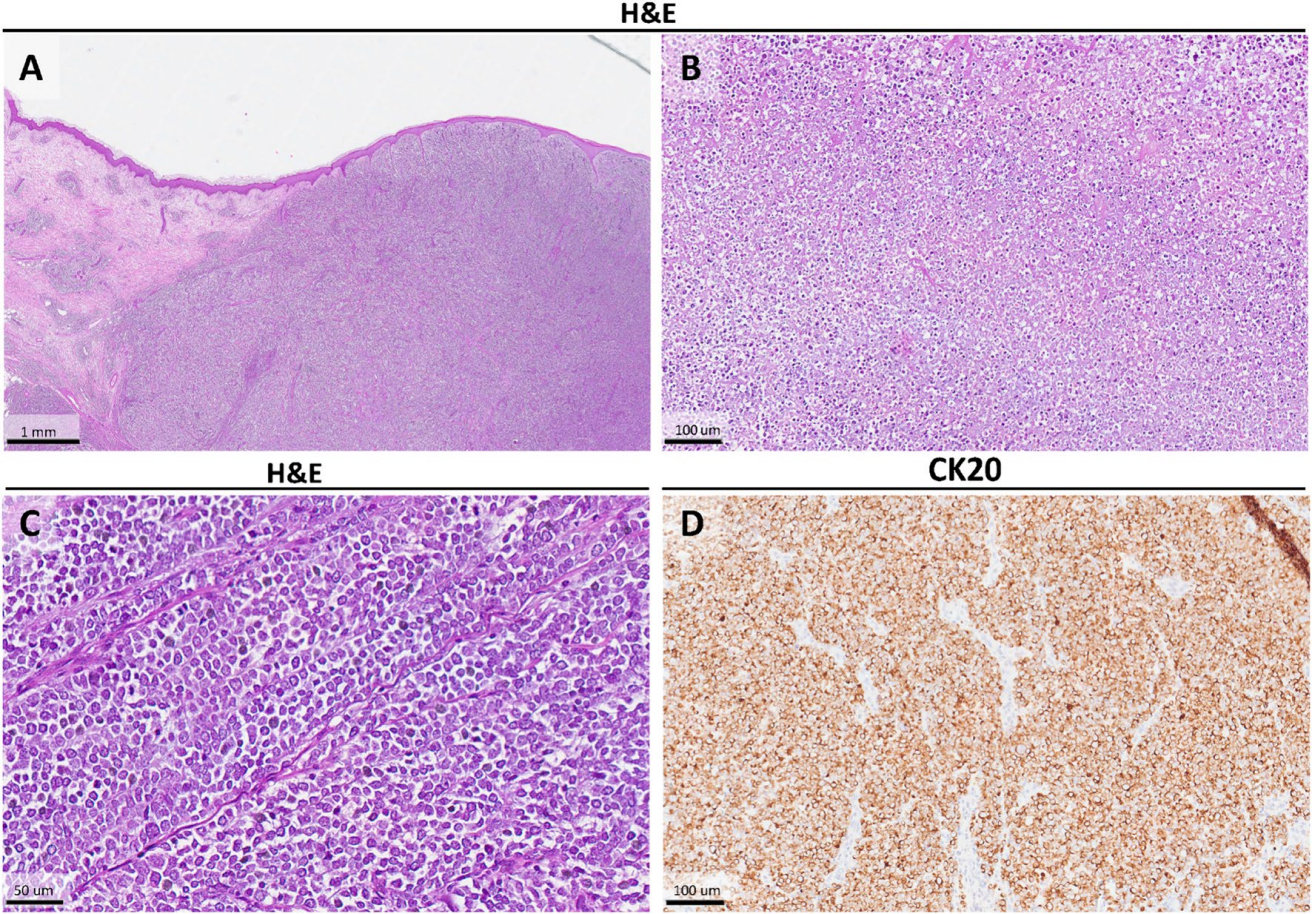
**a** View (4×) of a case of MCC: note the ‘blue tumour’ in the dermis and subcutis (Hematoxylin–Eosin, Original Magnification 4×). **b** Histopathological photomicrograph showing a necrotic area within MCC (Hematoxylin–Eosin, Original Magnification 40×). **c** Histopathological photomicrograph in which it’s possible to appreciate the ‘salt-and-pepper’ chromatin of the nuclei of the MCC cells (Hematoxylin–Eosin, Original Magnification 20×). **d** Immunohistochemical preparation for anti-CK20 antibody: note the typical ‘dot-like’ positivity of this marker that is a reliable proof of diagnosis of MCC (Immunohistochemistry for anti-CK20, Original Magnification 10×)

Histopathologically, the tumor presents as so-called blue nodule in the dermis and/or subcutis and consists of cellular elements that may be small or intermediate to large cells, the nuclei of which characteristically display the fine granular 'salt-and-pepper' appearance of chromatin, with frequent nuclear molding [2]. An example of histopathological features is reported in Figure 1a–c. In terms of immunohistochemistry, MCC cells are positive for epithelial markers such as CK-CAM5.2, CKAE1-AE3, CK20, CK34BetaE12, BerEP4, and Epithelial membrane Antigen (EMA), PIEZO2 [9], with some cases showing positivity for CK7 along with markers of neuroendocrine differentiation including chromogranin A (CgA), synaptophysin (Syn), and CD56 (NCAM1). It is also important to mention that positivity for CK20 is defined as 'dot-like' and is a reliable specific sign for the diagnosis of MCC [10, 11]. An example of CK20 is reported in Fig. 1d. The recurrence rate of MCC is about 40% [12] and regional spread occurs in 55% of patients with distant metastases occurring in about 35% of patients and affecting liver, bone, lungs, and skin [12–14].

It is now quite clear in the literature that mast cells play a particularly important role in the tumor microenvironment (TME). Both mast cells and macrophages exhibit regulatory effects in the context of both inflammation and immunological response. Increased numbers of mast cells have been shown to correlate with a poor prognosis in numerous solid and haematological cancers, but, on the other hand, in some cancer settings they may also exert an anti-tumor effect by eliciting cancer rejection [14]. Growth factors derived from mast cells that can promote tumour development and angiogenesis include fibroblast growth factor (FGF)-2, tryptase, chymase, osteopontin and nerve growth factor (NGF), vascular endothelial growth factor (VEGF), platelet-derived growth factor (PDGF), interleukin (IL)-8 and IL-10 (high expression) (angiogenesis and tumor growth), and histamine through H1 receptors. In contrast, mast cells inhibit tumor growth, by releasing IL-1, IL-2, IL-4, IL-6, IL-10 (low expression), monocyte chemotactic protein-3 and -4 (MCP-3 and -4), histamine through H2 receptors, interferon alpha (IFNα), transforming growth factor (TGF)-β, tumor necrosis factor (TNF)-α, and leukotriene B4 (LB4) [15]. Among the various proteases that are secreted by mast cells, an important role is played by tryptase, which can stimulate endothelial cell proliferation and vascular tube formation in *in vitro* models. Therefore, the expression of tryptase, as well as chymase, has been shown to correlate with angiogenesis during cancer progression [16, 17].

Recently, it is underscored the importance of the immune response in MCC; for example, (according to [18]) CD8+ lymphocytes were found to significantly influence overall survival and disease-specific survival; furthermore, it was demonstrated that MCPyV presence serves as a strong prognostic factor, triggering a host immune response involving various lymphocyte subclasses such as CD3, CD8, FoxP3, and PD-L1 positive cells in MCC. Through the use of mass cytometry and combinatorial tetramer staining, researchers [19] have found that the baseline frequencies of MCPyV-specific cells in the blood are correlated with treatment response and overall survival. Interestingly, these cell frequencies decrease significantly during the response to therapy and the phenotypes of MCPyV-specific CD8 T cells exhibit distinct expression patterns of CD39, cutaneous lymphocyte-associated antigen (CLA), and CD103. Finally, another study demonstrated that the MAGE-A3 antigen, frequently expressed in MCC, was recognized by CD8 TILs obtained from a virus-negative MCC tumor, suggesting that MAGE-A3 could potentially serve as a target for immunotherapy in virus-negative MCC case [20].

Until now, one study [21] analyzed the prognostic role of mast cells in MCC, finding a relationship between mast cell density and worsening prognosis. In this paper, we conduct a descriptive study of the density and spatial distribution of mast cells in 13 MCC specimens belonging to as many patients, describe the clinical (age, location, comorbidities, timing at diagnosis) and histopathological (maximum diameter, lymphovascular invasion, recurrence/metastasis/death, and distance from the nearest margin) characteristics, and establish a possible relationships between these parameters.

## Materials and methods

### Processing methods

In this retrospective investigation, paraffin-embedded blocks were retrieved from the archives of Pathology Department, University of Bari “Aldo Moro”, Italy, for all MCC identified on a computer search over a 20-year period (2003–2023 inclusive), with 13 cases recovered. Informed written consent from each patient was obtained prior to the study's conduct, and all procedures were carried out in accordance with the Helsinki Declaration of 1964 and later versions, as well as the ethical standards of the responsible committee on human experimentation (institutional and national). The original haematoxylin–eosin sections were re-evaluated in double-blind by two dermatopathologists (G.C. and A.C.) and any immunohistochemical staining performed. When the preparations had undergone significant deterioration, new sections in EE and/or IHC were re-mounted. All reviewed cases had diagnostic concordance and once the re-evaluation process was completed, new sections were cut by immunostaining with anti-triptase (Monoclonal Mouse, Anti-Human Mast Cell Tryptase, Clone AA1, 1:100 dilution, Dako-Agilent), anti-CD34 (Monoclonal Mouse Anti-Human, Clone QBEnd 10, 1:100 dilution, Dako-Agilent), and anti-MCPyV (large T-antigen, Clone CM2B4, 1:200 dilution, Gennova) antibodies. Detailed clinical information of the patient such as age at diagnosis, localization, comorbidities, timing at diagnosis (months) and histopathological information reported on the reports and appropriately supplemented when absent, such as maximum macroscopic diameter of the lesion (mm), lymphovascular invasion (LVI), recurrence/metastasis/death, and distance from the nearest margin (mm) were retrieved. All cases had a follow-up of at least 24 months.

### Imaging analysis

The sections from each patient were scanned using the whole-slide (WSI) morphometric analysis scanning platform Aperio Scanscope CS (Leica Biosystems, Nussloch, Germany). All the slides were scanned at the maximum available magnification (40×) and stored as digital high-resolution images on the workstation associated with the instrument. Digital slides were inspected with Aperio ImageScope v.11 software (Leica Biosystems, Nussloch, Germany) at 20× magnification and ten fields with an equal area were selected for the analysis at 40× magnification. The protein expression was assessed with the Positive Pixel Count algorithm embedded in the Aperio ImageScope software and reported as positivity percentage, defined as the number of positively stained pixels on the total pixels in the image.

### Statistical analysis

To evaluate whether mast cell density was different in relation to recurrence/metastasis/death (yes/no) and lymphovascular invasion (yes/no), the Mann Whitney U test was performed. A similar analysis was performed to evaluate whether vascular density (CD34+) was different in relation to recurrence/metastasis/death (yes/no) and lymphovascular invasion (yes/no). The choice of a non-parametric test was due to the limited number of observations. Fisher Exact test was used to evaluate the association between recurrency/metastasis/death and MCPyV status.

## Results

### Case series

The study group consisted of nine males (69.2%) and four females (30.7%) with a mean age of 73.2 years; seven patients had a lower limbs lesion, three patients had a facial lesion (including one eyelid MCC) and three patients had an upper limbs lesion. The mean timing at diagnosis (time elapsed from the initial biopsy to the time of excisional biopsy) was 4.3 months (range 0–12), and the mean maximum diameter was 37.3 mm (range 12–111).

Of the 13 patients included in the study, three (23%) died directly from Merkel cell carcinoma. Four (30.7%) developed a metastatic tumour and two (15.3%) had a recurrence at the site of the original surgery. These two patients had tumors extending to, or within 0.5 mm from the deep and/or lateral margin. 30.7% of the group (four patients) suffered neither recurrence, metastasis nor mortality as a direct consequence of Merkel cell carcinoma. seven patients (53.8%) received chemotherapy. Each patient had a follow-up of 24 months (2 years). Patients who received chemotherapy had tumors with a larger mean diameter (21.0 mm) than those who did not receive chemotherapy (14.5 mm). Two patients had neoplastic comorbidity Chronic Lymphocytic Leukemia (CLL). Some important clinical features are reported in Table 1.

**Table 1:** Clinical features in our cohort of 13 MCC patients

	Number of patients (%)
Tumor recurrence at the site of previous MCC	2 (15.3)
Metastasis	4 (30.7%)
Death from MCC	3 (23%)
No metastasis/recurrence/death from tumor	4 (30.7%)

### Histopathological features

Histopathologically, regarding LVI, 7 patients (53.8%) have LVI while six patients (46.1%) did not have LVI.

All MCC cases (100%) were positive for CK20 in the typical dot-like pattern.

### Immunohistochemical features

**Fig. 2:**
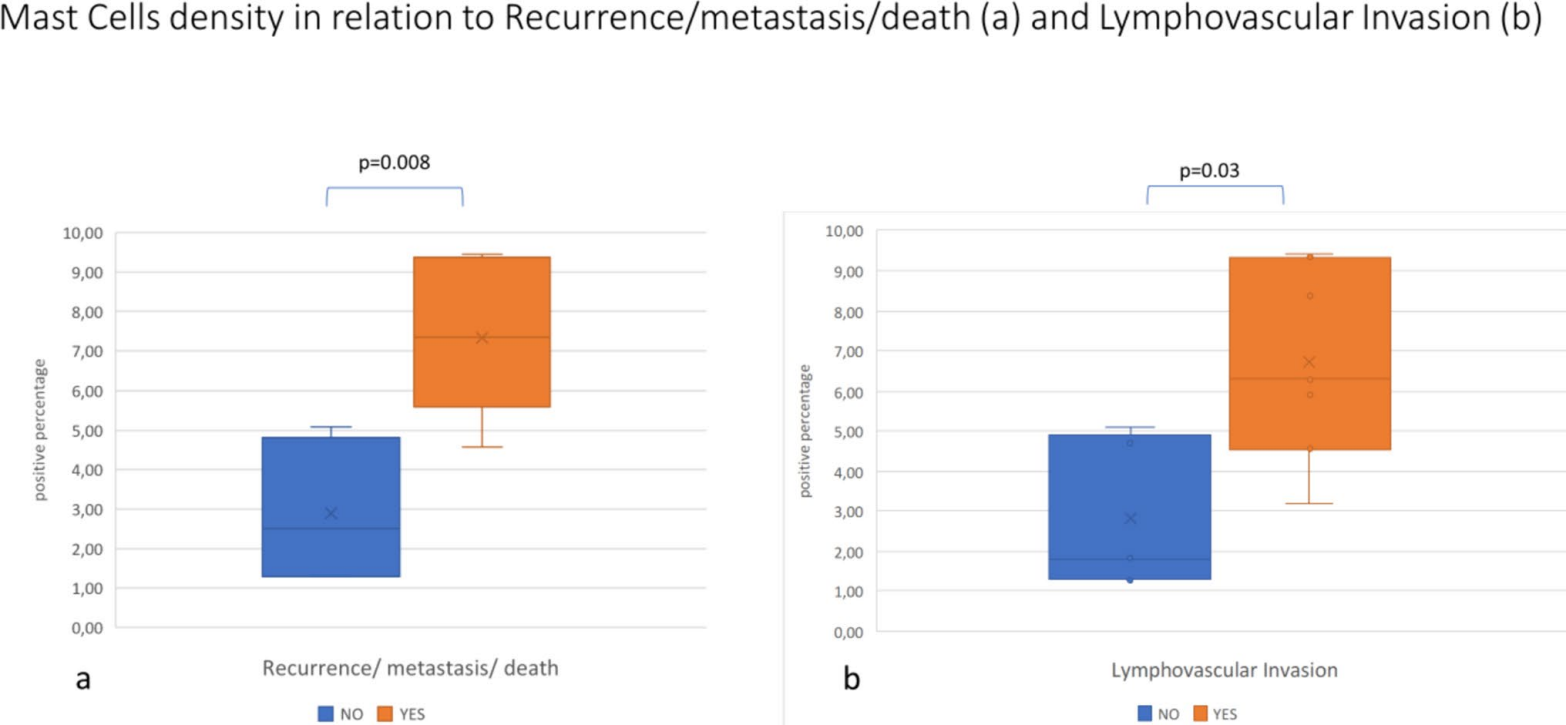
Mast cells density in relation to recurrency/metastasis/death (**a**) and lymphovascular invasion (**b**)

The distribution of mast cells density in relation to recurrence/metastasis/death and in relation to lymphovascular invasion is shown in Fig. 2, in sections a and b, respectively.

The median mast cell density observed on excisional biopsy of patients with recurrence (or metastasis/death) is significantly higher than the median observed on excisional biopsy of patients without recurrence (or metastasis/death) (7.35 vs 2.5; *p* = 0.008) (Fig. 2a).

The same significant difference was observed in the median mast cell density regarding the presence or absence of lymphovascular invasion (6.31 vs 1.82; *p*=0.03).

Of the seven patients (53.8%) who had died or developed metastatic disease all had histologically determined lymphovascular invasion and had the highest mast cell density in the TME.

**Fig. 3:**
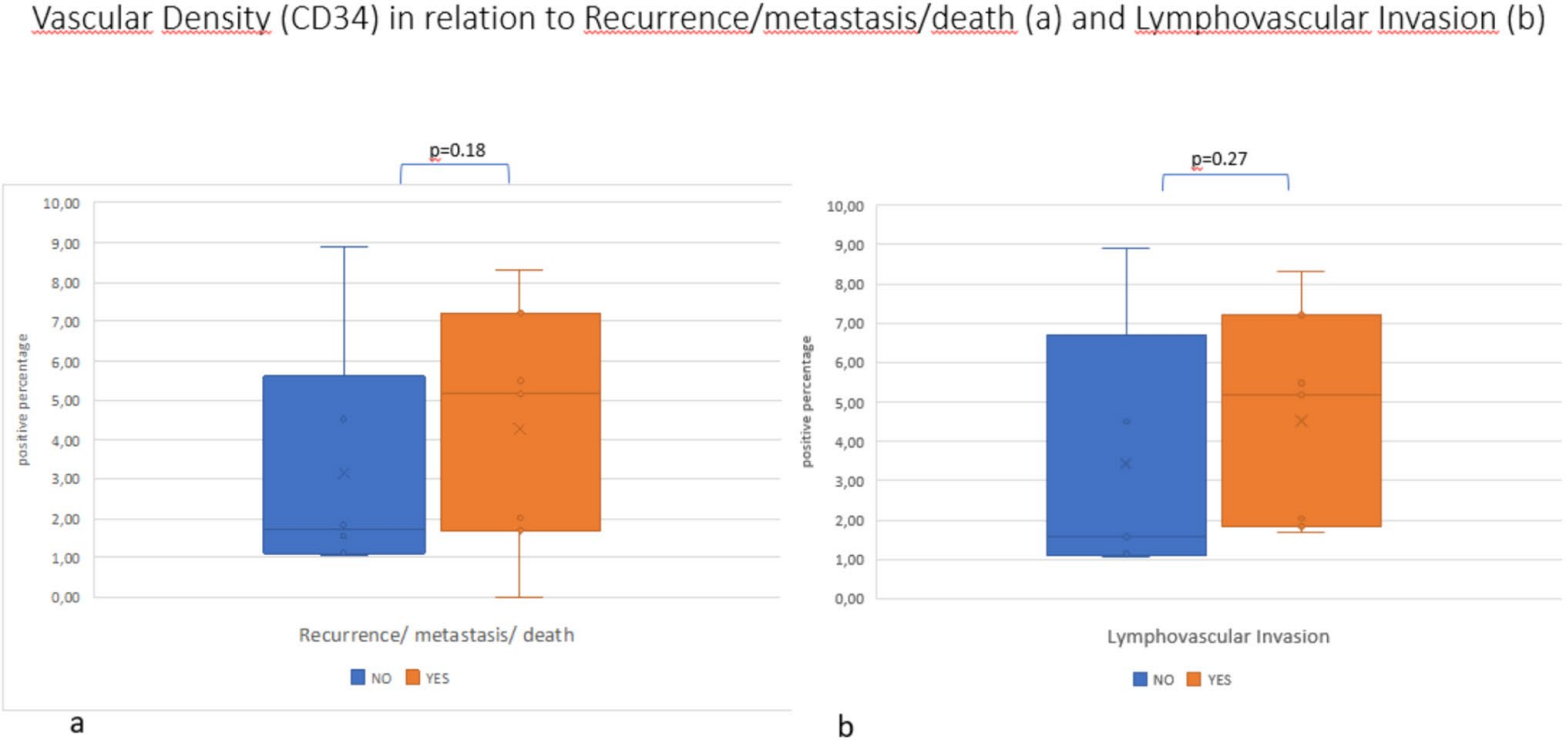
Vascular density highlighted by CD34 in relation to recurrency/metastasis/death (**a**) and LVI (**b**): note the absence of statistical significance (*p* = 0.18 and *p* = 0.27, respectively)

The distribution of vascular density in relation to recurrence (or metastasis/death) and in relation to lymphovascular invasion is shown in Fig. 3, in sections a and b, respectively. No significant difference was observed between the median vascular density observed on excisional biopsy of patients experiencing recurrence (or metastasis/death) and the median observed on excisional biopsy of patients without recurrence (5.3 vs 1.7; *p* = 0.18) (Fig. 3a). No significant difference was observed in the median vascular density regarding the presence or absence of lymphovascular invasion (5.18 vs 1.57; *p*=0.03).

Regarding MCPyV status, 8/13 patients (61.5%) were negative for MCPyV, while the remaining 5/13 patients (38.4%) were correlated to Polyomavirus.

**Fig. 4:**
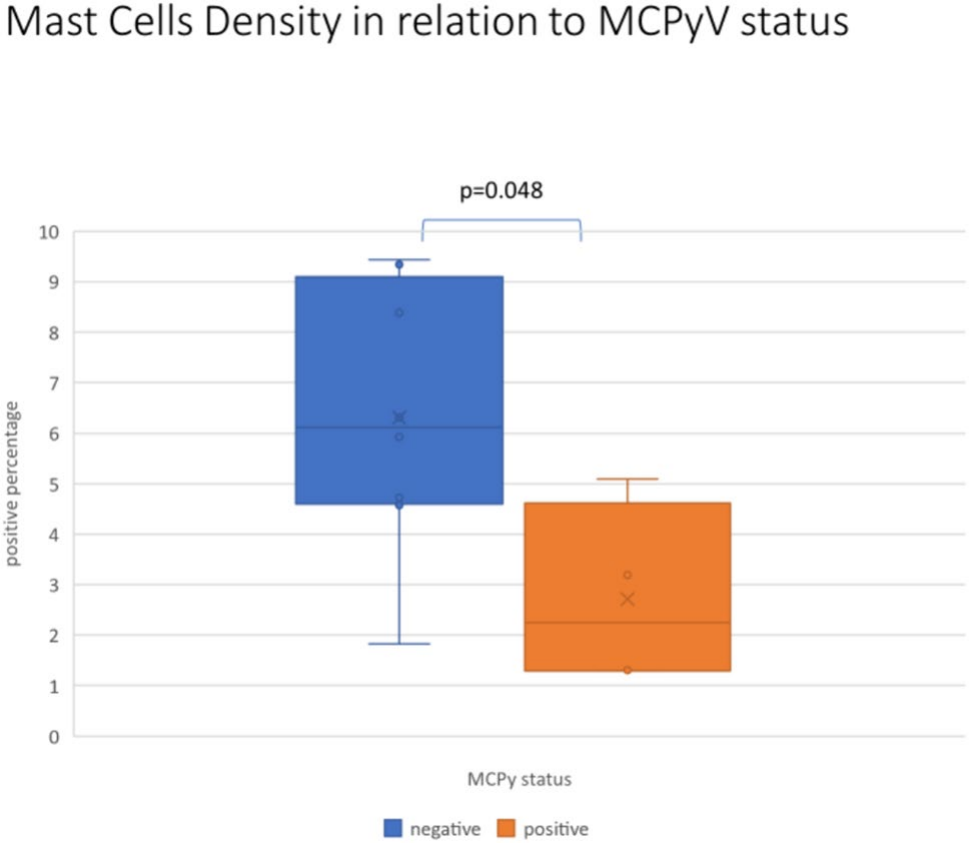
Mast cells density in relation to MCPyV status of MCC: note the statistically significance between the two groups (*p* = 0.048)

The distribution of mast cell density in relation to MCPyV status is shown in Fig. 4.

The median mast cell density observed on excisional biopsy of patients with MCPyV negative status is significantly higher than the median observed on excisional biopsy of patients with MCPyV positive status (6.1 vs 2.2; *p* = 0.048) (Fig. 4). Furthermore, MCPyV status is associated with recurrence (or metastasis/death), in fact none of patients with recurrence or metastasis resulted positive to MCPyV respect 67% of patient without recurrence (or metastasis/death) (0 vs 67%, Fisher Exact Test *p* = 0.03).

**Fig. 5:**
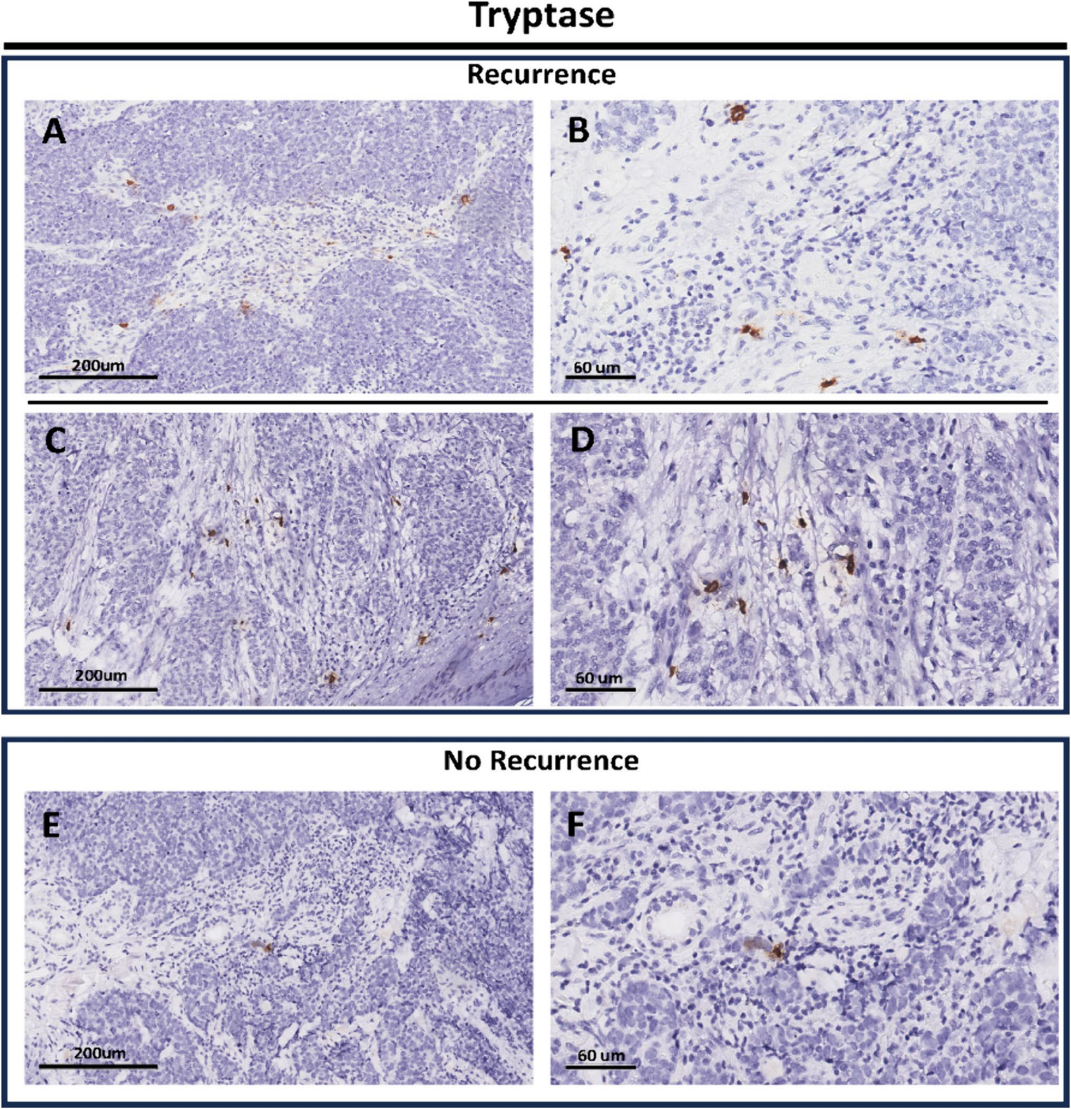
**a** Immunohistochemical preparation for anti-Tryptase antibody: note the presence of scattered mast cells highlighted by brown chromogen (Diaminobenzidine, DAB) in the peritumoral and intratumoral microenvironment of a case of MCC with subsequent metastasis (Immunohistochemistry for anti-Tryptase, Original Magnification 20x). **b** Another field of the previous case showing some mast cells in the TME (Immunohistochemistry for anti-Tryptase, Original Magnification 40x). **c** Another case of MCC with subsequent recurrence (Immunohistochemistry for anti-Tryptase, Original Magnification 20x). **d** Scanning magnification of the previous picture showing some mast cells in the TME (Immunohistochemistry for anti-Tryptase, Original Magnification 40x); **e** An example of MCC that at follow-up of 24 months had not given recurrence or metastasis: note the few mast cells present in the TME (Immunohistochemistry for anti-Tryptase, Original Magnification 20x); **f** Scanning magnification of the previous image showing only one mast cells in TME (Immunohistochemistry for anti-Tryptase, Original Magnification 40x)

**Fig. 6:**
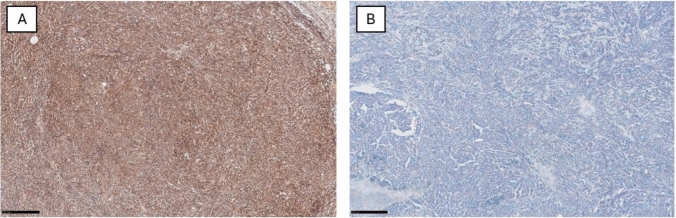
**a** Immunohistochemical preparation for anti-MCPyV antibody: note the diffuse positivity in this case of MCPyV-positive MCC (immunohistochemistry for MCPyV antibody, original magnification 10×). **b** Negative control of another case of MCPyV-negative MCC (immunohistochemistry for MCPyV antibody, original magnification 4×)

Examples of spatial and density distribution of peritumoral and intratumoral mast cells are reported in Fig. 5a–f and examples of positive/negative MCC for anti-MCPyV antibody are presented in Fig. 6a–b.

## Discussion

MCC is a rare, highly aggressive, neoplasm with sometimes higher morbidity, mortality, and recurrence/metastasis rates than malignant melanoma (MM) [12,22, 23]. Its clinical diagnosis is particularly difficult as there are still no reliable dermoscopic criteria to differentiate it from more common primary cutaneous neoplasms such as basal cell carcinoma (BCC), squamous cell carcinoma (SCC), and MM [21]. Histopathologically, diagnosis is not particularly complex unless there is the possibility of loss of immunohistochemical expression of commonly present markers including CK-20 with 'dot-like' pattern [24]. Several studies have tried to investigate the presence of mast cells in the tumor microenvironment of MM and non-melanoma skin cancer (NMSC), such as BCC, SCC, and Kaposi's Sarcoma [25, 35, 36]. Concerning MM, it was first shown in 1980 [25] that the number of perivascular mast cells was increased in the TME of cutaneous melanoma and a subsequent study [26] correlated the density of mast cells with vascular density and found a positive correlation between VEGF-positive mast cells and tumour microvessels. It is interesting to note that a prospective study on the predictive significance of microvessels and mast cells in human melanoma has shown that the count of these was significantly higher in patients who had developed local lymph node metastases than in patients who were alive and free of metastases, and even higher in patients who had died [27]. In a study by Rajabi et al. [28], the distribution of mast cells in the intratumoral and peritumoral microenvironment of 51 patients with MM was studied; the authors reported a mean ± standard deviation (SD) of intratumoral mast cells in stages 1, 2, and 3 of MM of 9.4 ± 4.2, 10.8 ± 5.1, and 2.1 ± 2.3 with a *p* = 0.000. Furthermore, the mean ± standard deviation (SD) values of mast cells in the peritumoral microenvironment were 13.4 ± 2.4, 16.6 ± 2.4, and 8.2 ± 4.6 (*p* = 0.000) in stages 1, 2, and 3. The authors described, therefore, a significant direct relationship between the depth of the tumor and the lower presence of tumor-infiltrating lymphocytes (TILs) and distribution of mast cells, suggesting a possible inhibitory effect of infiltrating mast cells and lymphocytes on the progression of this tumor.

About MCC, however, very few studies to date have attempted to establish the role that mast cells play in prognostic terms. The main study that demonstrated a prognostic role of mast cells in MCC is from Beer et al [21][24]; the authors analyzed a cohort of 36 patients with MCC by assessing the immunohistochemical expression of tryptase, as we did. Their results indicated that an increased infiltration of mast cells in the TME was an independent prognostic factor worsening the diagnosis, as was the presence of LVI, a parameter already widely present in the MCC reporting guidelines [29]. Our study, despite the limited number of cases due to the rarity of the neoplasm, is, to the best of our knowledge, the second paper that has investigated this aspect, supporting the data previously reported by the above-mentioned authors. Importantly, we found a statistically significant relationship both between mast cell density and LVI (*p* = 0.03) and between mast cell density and risk of recurrence/metastasis or death from MCC. On the other hand, taking in account the importance of mast cells in directing events such as angiogenesis, immunosuppression, extracellular matrix degradation, and mitogenesis, we correlated vascular density (assessed by CD34) and the risk of LVI and/or recurrence/metastasis/death but found no statistically significant results. However, it is likely that this figure may be affected by the low sample size (*n *= 13) rather than it being possible to assume that there is no real relationship between these variables. Indeed, Beer et al. [21] pointed out that, due to the rarity of the neoplasm, the studies in the literature have a rather limited cohort of patients, with quite conflicting results; for example, larger tumor size (diameter max) has been found to be an adverse prognostic factor in some studies [30–32] but not in others [32–34]. Accordingly, we found no statistically significant relationship between maximum neoplasm diameter (mm) and prognosis (results not shown).

Another important aspect we addressed in our paper relates to the status of MCPyV; the current literature is in agreement that MCPyV-positive cases have a more favorable prognosis than non-MCPyV-related cases [35–37]. Different hypotheses have been advanced to explain these data, including the fact that MCPyV-negative MCCs have a higher number of chromosomal aberrations [8, 39, 40], a greater nucleotide mutation burden [8, 38], and a higher number of mutations in known oncogenic pathways including PI3K/pAKT, P53, and RB1 [8, 41], as well as postulating that virus-driven oncogenesis is able to be more immunogenic and thus stimulate a more vigorous response from the immune system [41, 42].

Last but not least, these results are consistent with previous studies that emphasized the presence of CD8+ lymphocytes and tumor-associated macrophages (TAMs) in the TME of MCC, suggesting that mast cells do not belong to the so-called shared oncogenic pathways common to MCPyV (+) and (−) MCC.

We fit into this groove with our results, which, although with a limited cohort of patients, demonstrated a different mast cell density between MCC-negative and MCPyV-positive cases. This statistically significant difference, to the best of our knowledge, has never before been reported in the literature and should be confirmed in the future with larger sample size studies as it contains enormous potential, also from a therapeutic point of view.

## Limitations

The most important limitation regarding our study is the sample size (*n* = 13), which, considering the prevalence of MCC in Europe (0.13 cases per 100,000 persons per year), is, in our opinion, acceptable but it should be emphasized that statistical analysis must be interpreted taking this limitation into account.

## Conclusions

Overall, we feel it is correct to state that reporting in the histopathological report of MCC, in addition to the data that are commonly mandated, also includes a semi-quantitative assessment of mast cell infiltration, so that the clinician can be provided with additional information. Future studies with a higher sample size than ours are necessary to definitively confirm the prognostic value of mast cells in MCC.
